# Multiagency safeguarding arrangements during and beyond the Covid‐19 pandemic: Identifying shared learning

**DOI:** 10.1002/car.2774

**Published:** 2022-06-15

**Authors:** Jenny Driscoll, Aisha Hutchinson, Ann Lorek, Christopher Stride, Katrina Kiss

**Affiliations:** ^1^ King's College London England UK; ^2^ University of Sheffield England UK

**Keywords:** child protection, child safeguarding practice review panel, Covid‐19 pandemic, multiagency collaboration

## Abstract

Measures to combat transmission of the coronavirus presented unprecedented challenges for safeguarding and child protection practice, including through withdrawal of routine opportunities to observe and engage with children and families and disruption of systems for inter‐agency communication and coordination. This article reports on a two‐stage study designed to identify shared learning from adaptations to professional practice in response to the measures. Interviews with 67 London‐based senior safeguarding leads from seven professional groups undertaken during the summer of 2020 informed an England‐wide survey to similar groups in February–March 2021. SPSS was used to analyse 417 responses, which were supplemented by answers to open questions. Findings are reported using the six practice themes which the Child Safeguarding Practice Review Panel expects to inform shared learning to improve safeguarding at national and local levels. The study revealed the formidable barriers facing professionals in understanding the changing environments in which children were living and in identifying and assessing new or altered risks due to the pandemic; steps taken to respond to changing risks and to keep in touch and re‐engage families; strategies to support critical thinking and challenge among professionals working under unprecedented pressure; and opportunities for enhanced multiagency working and inter‐agency collaboration.

**Key Practitioner Messages**

- The challenges appear to have fostered an enhanced commitment to inter‐agency collaboration.
- The pandemic has highlighted the importance of an organisational culture that attends to staff well‐being, supervision and support.
- Online early help services may place greater onus on families to take the initiative to contact services, raising concerns as to accessibility and evasion of professional scrutiny.
- Families appeared to experience ‘keep in touch’ strategies as more supportive and less threatening than professional involvement under normal circumstances.

## INTRODUCTION

The importance and challenge of inter‐agency communication and collaboration has been a persistent theme in English child protection work over many years, evidenced in biennial/triennial meta‐analyses of serious case reviews (most recently Brandon et al., 2020; Sidebotham et al., 2016). The policy response to this challenge has been to require coordination of child safeguarding between the agencies involved at local area level, with statutory duties on these agencies to cooperate with inter‐agency arrangements (Children Act 2004) and statutory guidance to promote inter‐agency working (currently Department for Education (DfE), 2018). Within each agency, designated professionals for safeguarding provide a contact point, support and advice for colleagues and/or liaison with other agencies. Following the Wood Review (Wood, 2016), which concluded that the previous arrangements were insufficiently robust, Local Children's Safeguarding Boards, led by local authorities, were replaced by Safeguarding Partnerships in local government areas (Children and Social Work Act 2017). Under the new arrangements, local authorities, National Health Service Clinical Commissioning Groups and the police (the ‘Safeguarding Partners’) became jointly accountable for making arrangements to work together and with any ‘relevant agencies’ (such as schools) that they consider appropriate to identify and respond to the needs of children in their area.

The same legislation replaced serious case reviews with a system of rapid reviews and local child safeguarding practice reviews and introduced greater national oversight through establishment of the Child Safeguarding Practice Review Panel (CSPRP, hereafter ‘the Panel’). The Panel has to date published two annual reports, covering mid‐2018 to the end of 2019 (Child Safeguarding Practice Review Panel (CSPRP), 2020) and 2020 (Child Safeguarding Practice Review Panel (CSPRP), 2021). In the latter, the Panel identified six key practice themes arising from analysis of evidence from incidents notified to the Panel by Safeguarding Partnerships, rapid reviews and local safeguarding children practice reviews. These are: understanding what the child's daily life is like; working with families where their engagement is reluctant and sporadic; critical thinking and challenge; responding to changing risk and need; sharing information in a timely and appropriate way; and organisational leadership and culture for good outcomes (2021, p. 7). The last theme is described as underpinning all the others. While the themes are not purported to reflect all aspects of good practice, the Panel believes they will contribute to reducing serious harm and preventing deaths. The Panel expects the themes to inform shared learning through Safeguarding Partnerships to improve safeguarding at local and national levels.

Local areas were required to have implemented the transition to Safeguarding Partnerships approximately six months before the first national ‘lockdown’ to delay the spread of Covid‐19 was imposed at the end of March 2020. The strict lockdown measures, including ‘stay at home’ guidance, closure of schools to most children and widespread withdrawal of services, presented unprecedented challenges for safeguarding practice. A number of commentators, including the Children's Commissioner (2020, p. 27), expressed concern that children were ‘side‐lined’ by the measures to combat the pandemic. Although during periods of lockdown schools were open for children identified as ‘vulnerable’ under government guidance (DfE, 2020a), attendance by these groups was low, particularly early in the pandemic (Romanou & Belton, 2020). The restructuring of health services in response to the influx of Covid‐19 patients resulted in significant redeployment of frontline universal practitioners (Adams, 2020; Conti & Dow, 2021; Evans, 2020; Institute of Health Visiting [IHV], 2020), who are the source of a considerable proportion of referrals to children's social care services (DfE, 2020b). Redeployment of safeguarding health staff likewise disrupted the pathways for strategic oversight, inter‐agency communication and supervision (Green, 2020).

The ‘stay home’ guidance also resulted in reduced attendance at hospital emergency departments and for paediatric assessments (Crawley et al., 2020; Lynn et al., 2020). Fewer opportunities for staff to interact with children and identify safeguarding concerns are evidenced by an average 10 per cent fall in the number of referrals to children's social care services from the end of April 2020 to mid‐July 2021 (DfE, 2021). In contrast, serious incident notifications to the Panel increased by 19 per cent in the year April 2020–March 2021, compared with the preceding 12 months (Department for Education (DfE)/Office for National Statistics (ONS), 2021), and by 8 per cent compared with 2018–19. These data are in line with international evidence of reduced referrals to police and child protective services during the Covid‐19 pandemic, alongside increased family violence and hospital treatment for child‐abuse related injuries (Cappa & Jijon, 2021). At the time of writing, results from enquiries into the deaths of Arthur Labinjo‐Hughes and Star Hobson are awaited, but it appears likely from media reports that children dropping from professional view, increased family stress and/or staffing pressures during lockdown periods were implicated in their deaths.

This article reports on a study designed to identify shared learning from adaptations to professional practice in response to the measures. The term ‘safeguarding’ encompasses both the formal child protection system, through which statutory duties to protect children who may be at risk of significant harm are enacted, and a broader collaborative framework for the promotion of child welfare, the prevention of maltreatment and professional support for families. Following explanation of the study methodology, this article analyses the study's findings using the Panel's six themes and considers the implications for multiagency working and inter‐agency collaboration.

## METHODS

The project was conceptualised as a two‐part modified Delphi study, a model characterised by the repeated consultation of experts to capture emerging areas of consensus (Iqbal & Pipon‐Young, 2009), and widely used in emerging areas of health and social care research (Hackett et al., 2006). It is therefore well suited to the research aim of identifying shared learning from adaptations to professional practice in response to measures imposed to combat the spread of the pandemic. An initial qualitative interview round sought participants' accounts of key concerns in their professional arena and practice responses to those. Findings from this round informed the areas of focus and appropriate response options for questions in the second‐stage survey, to ascertain which initiatives reported by interviewees were the most widely employed and how effective they were considered to have been. The interview stage was confined to London, but in response to feedback that patterns of deprivation, service use, structure of services and multiagency arrangements vary across regions, the survey was distributed throughout England.

Ethical approval was granted by King's College London Research Ethics Committee. Stage 1 was deemed a service evaluation by participating NHS organisations. NHS ethical approval was not required for stage 2 because no respondents were contacted directly through the NHS. At the time of the study, the Association of Directors of Children's Services had temporarily suspended oversight of research in multiple children's services departments. Each local authority was contacted individually through the Director of Children's Services. Due to the seniority of many participants and local‐level variants in titles, given professional roles have been truncated where necessary to ensure anonymity.

In the first stage, completed between June and early September 2020, 67 semi‐structured interviews were undertaken with senior safeguarding leads in children's social care, health, police, education and mental health services, lawyers with child representation accreditation, and Safeguarding Partnership independent scrutineers or chairs (Table 1). During this period, the country was under partial Covid‐19 restraints following the phased reopening of schools from 1 June 2020.

**TABLE 1:** Interview participants

Profession and designation	No. of participants (boroughs represented)	Positions held by interviewees
Children's Social Care I‐Children's Social Care‐1 to 11	11 (11)	5 directors of children's services
		5 assistant directors
		1 child protection conference chair
Health I‐Health‐1 to 15	15 (9) 3 acute hospital trusts	3 designated doctors
		8 designated nurses
		4 named doctors or nurses
Mental Health I‐Mental Health‐1 to 8	8 (14)	8 mental health safeguarding leads
Police I‐Police‐1 to 8	8 (19)	6 detective superintendents
		1 detective chief inspector
		1 detective inspector
Law I‐Law‐1 to 6	6	6 children's panel lawyers
Education I‐Education‐1 to 10	10 (11)	5 local authority directors of education/learning
		1 local authority schools officer
		4 head teachers involved in Safeguarding Partnership work
Safeguarding Partnerships I‐Safeguarding Partnerships‐1 to 9	9 (10)	8 independent chairs/scrutineers
		1 Safeguarding Partnership manager

The same groups were invited to complete the survey, with extension to Safeguarding Partnership business managers and local authority lawyers. An expert reference group comprising a representative from each respondent group piloted the survey and assisted with its dissemination through organisational networks. Senior safeguarding leads in each police area and Directors of Children's Services were contacted directly but may have forwarded the invitation to a nominee. The survey was hosted on Qualtrics survey software and was live from 1 February to 8 March 2021.

Responses were received from 563 respondents, from which an analysis sample of 417 was derived. Exclusions predominantly arose from failure to provide primary disciplinary affiliation, which affected filtering and analysis. Some questions were administered only to a sub‐group of respondents, resulting in fewer responses.

All regions of England were represented in survey responses, although 45% of respondents worked in London or the South East. Respondents from all professional groups contributed in each region, with the exception of mental health in the North West and West Midlands and police in the North East. Respondents' mean safeguarding experience was 16 years. Seventy‐two per cent reported involvement in the work of their local Safeguarding Partnership at some level. However, caution should be exercised in interpreting the findings in relation to the balance of disciplines and roles between and within groups. In particular, health professionals made up 37 per cent of the respondent group. Table 2 shows the distribution of respondents between professional groups and regions. It should be noted that professional roles in different disciplines are not equivalent and in some groups, such as health, a wide range of different roles are represented, while in others, particularly police and children's social care, professional roles are more uniform. It is not possible to identify response rates because of the means used to distribute the survey.

**TABLE 2:** Survey response by professional group and region

	Mental health	Health	Law	Education	Children's social care	Police	Safeguarding partnerships	TOTAL
**South East England**	2	31	8	10	12	3	4	**70**
	10.5%	20.0%	11.3%	17.9%	23.1%	10.0%	11.8%	**16.8%**
**South West England**	1	10	6	3	1	3	2	**26**
	5.3%	6.5%	8.5%	5.4%	1.9%	10.0%	5.9%	**6.2%**
**London**	9	38	28	13	14	6	8	**116**
	47.4%	24.5%	39.4%	23.2%	26.9%	20.0%	23.5%	**27.8%**
**West Midlands**	1	13	2	3	2	2	3	**26**
	5.3%	8.4%	2.8%	5.4%	3.8%	6.7%	8.8%	**6.2%**
**East Midlands**	0	10	7	16	3	2	3	**41**
	0%	6.5%	9.9%	28.6%	5.8%	6.7%	8.8%	**9.8%**
**East of England**	2	11	7	4	4	4	5	**37**
	10.5%	7.1%	9.9%	7.1%	7.7%	13.3%	14.7%	**8.9%**
**Yorkshire & The Humber**	3	21	6	1	2	9	4	**46**
	15.8%	13.5%	8.5%	1.8%	3.8%	30.0%	11.8%	**11.0%**
**North East England**	1	5	2	2	9	0	2	**21**
	5.3%	3.2%	2.8%	3.6%	17.3%	0%	5.9%	**5.0%**
**North West England**	0	14	5	4	5	1	3	**32**
	0%	9.0%	7.0%	7.1%	9.6%	3.3%	8.8%	**7.7%**
**National role**	0	2	0	0	0	0	0	**2**
	0%	1.3%	0%	0%	0%	0%	0%	**0.5%**
**TOTAL**	**19**	**155**	**71**	**56**	**52**	**30**	**34**	**417**
	100%	100%	100%	100%	100%	100%	100%	**100%**

Quantitative data were analysed using IBM's SPSS (Statistical Package for the Social Sciences) analytical software. Thematic analysis of answers to open questions confirmed key themes from interviews, including concerns about ‘hidden’ children, rapidly changing circumstances, challenges in risk assessment, information sharing and the impact of the conditions on practitioners. The close alignment of our analytical themes with the Panel's key themes and our overarching interest in multiagency leadership and coordination led to adoption of those themes in the findings presented here. In the following section, quotations are prefixed with I for interviewees or S for survey respondents.

## RESULTS

### Understanding what the child's daily life is like

The Panel's first theme aligns closely with the ‘child‐centred approach’ identified in the Munro review of child protection (Munro, 2011), one of two principles underpinning inter‐agency statutory guidance (DfE, 2018). It requires observing children's lives at first hand and forging trusting relationships with children and families.

Professionals' opportunities to build a picture of children's experiences were significantly reduced through the redeployment of frontline and specialist safeguarding professionals, and the withdrawal of practitioners from direct work with children and families in favour of remote interactions. Our survey revealed considerable strength of feeling regarding redeployment, with more than three‐quarters of respondents agreeing that safeguarding midwives, health visitors and designated safeguarding doctors and nurses should never be redeployed. Survey respondents endorsed interviewees' unease about the impact of the shift to remote working on professionals' ability to identify safeguarding concerns in the following ways: not being able to pick up non‐verbal cues so readily (97 per cent), uncertainty about whether others are in the room with a child (95 per cent), exclusion of families who lack access to the necessary technology (93 per cent), risk of misunderstandings (85 per cent) and challenges in building rapport and conveying warmth and support (80 per cent) (see also Baginsky & Manthorpe, 2020; Cook & Zschomler, 2020; Ferguson et al., 2021). Interviewees raised particular concerns about withdrawal of face‐to‐face contact by health visitors in the context of the vulnerability of newborn babies and new mothers whose social support networks were inaccessible. As I‐Children's Social Care‐9 cautioned:

we really do not understand what the experiences of some of these children are, you cannot really do meaningful work virtually to understand what a child is experiencing, because you need to be in that relationship building and be in there, see where they are living, pick up the new answers.

In response to low take‐up of school places for ‘vulnerable’ children and concerns about children with no current involvement with safeguarding services who may be newly vulnerable, a range of ‘keep in touch’ strategies were introduced. Principal among these was regular contact during termtime and, in most cases, also during school holidays: interviewees described anything from weekly to daily telephone calls to families depending on the degree of concern. Other strategies in order of most common employment were provision of food parcels, IT services to support children's online learning, in person ‘doorstep’ visits, provision of books and games, and follow‐up of non‐responsive families by children's social care services and/or police liaison. Notably, I‐Education‐7 observed that issues were unlikely to emerge through telephone calls and considered that ‘doorstep’ visits were much more likely to elicit or confirm concerns. Numbers answering this section of the survey are quite small (n = 61–79) because the questions were non‐applicable (and thus filtered out) for most groups, but the question response options arose from strategies described by interviewees.

Follow‐up of non‐responsive families by police liaison was a point of contention among interviewees, with some reporting that it worked well and others concerned it would be seen as threatening. In the survey, however, over half of respondents reported using this tactic and a similar proportion indicated that they would do so should similar circumstances arise in future, suggesting it was regarded as effective where employed.

Families appeared to experience ‘keep in touch’ strategies as more supportive and less threatening than professional involvement under normal circumstances according to our interview evidence, which could improve relationships (see also Ferguson et al., 2021). As I‐Education‐5 explained:

those families where they are used to school staff phoning them on a regular basis because their son or daughter is in trouble, and so the relationship is quite fraught between them and the school, now they are experiencing a phone call every day to say, ‘How are you? How's it going? Anything we can do to help?’ So they have now got a really positive relationship … those relationships have been repaired because of the amount of care that the schools have shown.

Sixty‐three per cent of survey respondents agreed that ‘keep in touch’ strategies improved relationships between families and schools and only 11 per cent disagreed. The effect appeared to be less strong for relationships between families and Children's Social Care, with 35 per cent agreeing, 18 per cent disagreeing and 47 per cent neutral. Respondents who indicated that relationships had improved were asked if those improvements had been maintained: all those responding to that question agreed that they had, at least in part. However, at the time of the survey schools were still not open to most children: these perceptions may have changed as children returned physically to school.

To gauge overall confidence on how well professionals could assess children's experiences, the survey asked how well respondents considered that the voice of the child was heard under the constraints of the pandemic at strategic and individual level. Fifty‐nine per cent considered that individual children's voices were less readily heard and 16 per cent that they were more readily heard. Children's social care respondents were more optimistic than others, with 30 per cent reporting that children were less readily heard and 35 per cent that they were more readily heard, perhaps reflecting more frequent communication with children and young people using virtual methods.

### Working with families with reluctant or sporadic engagement

This theme is particularly relevant to cases below the threshold for statutory intervention, when familial cooperation with agencies is voluntary. This aspect of safeguarding work depends on robust inter‐agency early help services, to ensure monitoring of progress and appropriate escalation. Despite being flagged for attention by the Munro review (Munro, 2011), this area remains a relative weakness of English practice (Hood et al., 2016; National Audit Office, 2019). Although 89 per cent of respondents noted increased early help needs, 39 per cent reported cuts to early help in favour of statutory services. Ninety‐four per cent stated that provision shifted online but only 53 per cent regarded those online services as effective and 68 per cent considered that online early help services placed a greater onus on families to take the initiative to contact services. Although these figures vary between agencies, they nonetheless raise concerns as to the accessibility of services and the extent to which such arrangements make it easier for families to evade professional scrutiny. They also have significant implications for the future. In I‐Education‐7's words:

I think we need to invest in really early help and assessment to try and connect with families that would have been referrals … the worry will be that we we end up with more children going up to that very top of the continuum. And actually, all we are doing is storing up more trouble for ourselves in the next 18 months.

**FIGURE 1:**
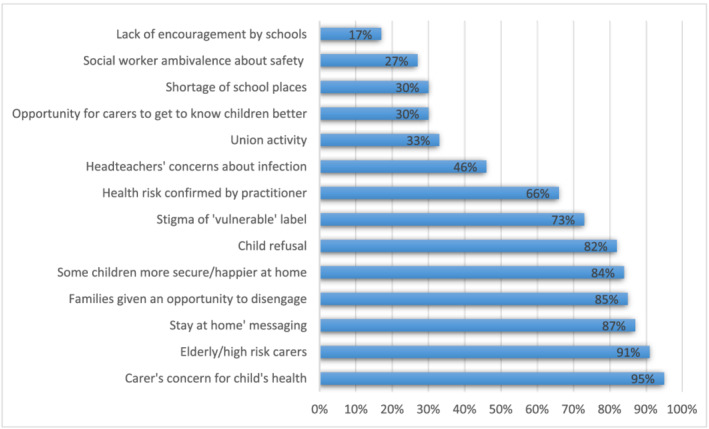
**Reasons thought to contribute to non‐attendance of vulnerable children** (number of valid responses varied from 79 to 130)

While recognising that there were many legitimate reasons for families to keep children entitled to a place out of school, using the pandemic as an opportunity to avoid the gaze of safeguarding professionals was a further area of concern. From interviewees' insights into the reasons given for some families' resistance to allowing their children to attend to school we compiled a list of response options in the survey (Figure 1). Although the first three related to concerns as to the child or carer's health and government ‘stay home’ messaging, ‘families given an opportunity to disengage’ was the fourth most cited option, with 85 per cent of those responding indicating that they considered it could contribute to non‐attendance. Agencies employed a range of strategies to try to boost attendance, with the most successful appearing to be encouragement of the school's Designated Safeguarding Lead or another member of school staff known to the family (Table 3).

**TABLE 3:** Strategies used to boost school attendance, and perceived effectiveness of strategies

	Used	Appeared to improve attendance to an extent/significantly
Encouragement of DSL/school staff known to family (74)	97%	83%
Using parental concerns about behaviour/schoolwork (71)	91%	68%
Encouragement of social worker (77)	87%	64%
Advice from health/mental health professionals (72)	75%	53%
Coordinated multiagency contact (72)	71%	51%
Virtual parents' group meetings (69)	52%	34%
Contact from School Improvement Service (74)	43%	23%
Youth mentoring (69)	43%	22%

Note that these items were only asked of respondents with knowledge of the involvement of schools in safeguarding arrangements during the pandemic. 159 respondents completed all or parts of that section of the survey, of which 35% were from Education. The number of valid responses to each item is given in parentheses. Column 2 gives the percentage of valid responses selecting ‘Used’. Column 3 gives the percentage of valid responses selecting ‘to an extent’ or ‘significantly’.

A further challenge to engagement, the detail of which is beyond the scope of this article, related to families' poor or limited access to technology or connectivity and the potential for them to blame technical problems for not communicating with professionals. I‐Health‐4, a designated nurse, summarised the challenge:

Particularly for families who do not really want to engage or disguise compliance. We've given them a licence to opt out … a big get out of jail free card … You've got digital poverty, they do not have IT access, they are not going online … how do you hold those children and get them back in the system?

### Critical thinking and challenge

‘Critical thinking and challenge’ relates to a lack of ‘professional curiosity’ and/or confidence in developing supportive but challenging relationships between professionals, including a reluctance to escalate concerns (CSPRP, 2021, p. 29). This reflects the second of Munro's principles (Munro, 2011) adopted in statutory guidance (DfE, 2018), that safeguarding is ‘everyone's business’, but goes further in drawing attention to the conditions that can impede critical thinking, including workload and weak supervision. Two factors appear particularly pertinent in the conditions imposed under the pandemic: the impact of the pressure under which professionals were working on their capacity for reflexive practice, access to reflective supervision and wellbeing, and the temporary suspension of training.

Seventy‐five per cent of survey respondents reported that the wellbeing of safeguarding professionals in their organisation had declined over the course of the pandemic from March 2020 to March 2021. They identified increased caring responsibilities, reduced staffing levels, increased workload and loneliness as the most common factors affecting staff wellbeing. Additional comments added the following insights: ‘High levels of sickness and continued anxiety impact on professional practice or professional curiosity’ (S‐Named Nurse for Safeguarding); ‘The health of the professionals working within this sector (social workers and Lawyers in my field) has been significantly impacted. Workloads are far higher with little to no respite. Furlough is not an option in the Local Authority’ (S‐Local Authority Solicitor).

Interviewees suggested that for some, particularly young professionals in children's social care and the police, who had previously endured lengthy commutes, these challenges were offset to some degree by the advantages of working from home – although poor home working conditions and loneliness were common and the withdrawal of informal sources of support were felt keenly. Compounding these challenges, interviewees reported that redeployment patterns reduced capacity for supervision and staff oversight as well as impacting on workload, communication and training. A range of strategies was used to support staff, most of which respondents considered to be helpful, particularly: regular contact with managers; opportunities for informal peer support; regular individual supervision; regular group supervision; and active management of leave, which were all rated as quite or very effective by at least 70 per cent of respondents.

Regular safeguarding training is imperative in ensuring professionals possess the skills to recognise and respond appropriately to risks confronting children and the confidence to challenge colleagues in their own or another discipline where necessary. Interviewees reported that training was predominantly suspended, with significant variation in organisations' capability to convert to online training swiftly. Survey respondents reported a significant shift in delivery, with 75 per cent describing training before the pandemic as all or predominantly in‐person, compared with only 2 per cent at the time of the survey, when 86 per cent reported training as all or predominantly carried out online. Yet only 2 per cent of respondents felt that training was best carried out online.

### Responding to changing risk and need

There is ample evidence from our study that, for many children, the ‘stay home’ guidance and changes in family composition, employment and financial status created more adverse home environments. Increased risk experienced by some children is evidenced by the agreement of survey respondents from all agencies that since the onset of the pandemic referrals had increased in both severity (84 per cent of respondents) and complexity (88 per cent). Exercises to reassess risk and prioritise needs were undertaken by children's social care in accordance with guidance from the Principal Child and Families Social Worker network and Social Work England (Buzzi et al., 2020), using a RAG (Red‐Amber‐Green) risk rating system. Interviewees related various approaches to risk reassessment, which were explored further in the survey. More than half of survey respondents (Law excluded) reported separate risk assessments by individual agencies, but 27 per cent reported some inter‐agency coordination and 20 per cent reported a single RAG rating exercise or equivalent coordinated by the Safeguarding Partnership. Regardless of the level of collaboration, most respondents (79 per cent) considered the exercise to be quite or very effective and only 9 per cent thought it was very or quite ineffective. Collaborative or shared assessment processes were more likely to be regarded as effective than separate risk assessments.

An important caveat was highlighted in qualitative comments from children's social care and health respondents, which was that the process did not enable identification of newly vulnerable children. Other comments highlighted concerns that the exercise did not result in action: ‘RaG rating is used to identify but nothing ever comes of it – no changes, no impact’ (S‐Education), or that failure to collaborate could exacerbate rather than ameliorate risks: ‘these ratings weren't shared – there was no discussion to understand those differences and that therefore created more risk’ (S‐Safeguarding Partnership Business Manager).

### Sharing information in a timely and appropriate way

A positive finding arising from other studies (e.g. Baginsky & Manthorpe, 2020; Ferguson et al., 2020), as well as ours, is the extent to which a shift to online communication and virtual meetings facilitated inter‐agency information sharing. While concerns around this practice are beyond the scope of this article, it is notable that interviewees and survey respondents reported better professional attendance and engagement at child protection meetings related to statutory processes such as child protection conferences.

Building on interviewees' descriptions of strategies introduced to promote information sharing in the context of concerns about rapidly changing levels of risk faced by children, survey respondents were asked if they had introduced, and if so, would support retention of, certain adaptations. The responses suggest that the pandemic heightened the perceived importance of inter‐agency communication and information sharing. Excluding ‘do not know’ responses, 70 per cent reported increased sharing of data and trends within their local authority area; two‐thirds reported increased scrutiny of data and trends at Safeguarding Partnership level; and just under a half (47 per cent) reported increased sharing of data and trends between local authority areas. There was strong support for retention of all these strategies. One‐third of respondents reported widening of the remit of the Multi‐Agency Safeguarding Hub (MASH) to encompass consideration of more groups of children: 83 per cent of these respondents considered that this model should be retained. Ninety‐eight per cent of respondents indicated that, from their experience during the pandemic, they would support introduction of a system by which all agencies could share pre‐agreed information relating to safeguarding children, with no agency returning less than 94 per cent support.

### Organisational leadership and culture for good outcomes

Recalling the immediate response to lockdown, our interviewees described an initial vacuum of decision‐making, provoking a sense that ‘everything stopped’ (I‐Health‐4). Agencies ‘hunkered down’ (I‐Safeguarding Partnerships‐1) as they tackled the immediate impact of lockdown on their own agency before re‐establishing inter‐agency connections. Survey respondents described decision‐making as ‘reactive’ (S‐Designated Nurse for Safeguarding) and ‘initially a knee jerk reaction’ (S‐Health), reflecting a view of safeguarding as ‘unimportant’ (S‐Named Midwife for Safeguarding Children) and ‘not prioritised by those in power’ (S‐Named GP for Safeguarding Children).

The extent to which safeguarding imperatives were understood and prioritised was reflected in redeployment decisions. While many of our research participants expressed dismay as staff in critical safeguarding roles were redeployed, several health professionals responding to the survey noted that staff were either not redeployed in their organisation or redeployment was limited, as the following example illustrates:

we increased Safeguarding supervision, provided a 7‐day Safeguarding help line for all staff. Issued clear directions promoting face to face visits to vulnerable children with PPE at a time that social workers were not doing statutory visits to CP children, this was due to effective leadership by our safeguarding director. (S‐Named Nurse for Safeguarding)

Decisions such as these reveal insights into the organisational culture of different agencies, including their appetite for risk, their prioritisation of safeguarding considerations, and how the interests of service users and staff are reconciled. The pandemic has also highlighted the importance of an organisational culture that attends to staff wellbeing and attaches importance to staff supervision and support, as poignantly demonstrated by a survey respondent:

I have had to seek out not only safeguarding supervision in my Trust but I have also asked our CAMHS team if they are able to support some reflective practice. Both have said they want to support me as a colleague but do not have capacity even remotely. This has left me feeling quite frustrated, unmotivated and low at some points. There has been pressure from senior management to physically be in an office where we are unable to socially distance and colleagues are not wearing face coverings despite being able to work from home. (S‐Named Nurse)

However, at multiagency level, the extraordinary challenges facing safeguarding professionals appear to have fostered an enhanced commitment to inter‐agency collaboration. Many interviewees conveyed a sense that ‘Covid brought everyone together’, while survey commentary included: ‘The relationships during the pandemic have led to decrease in bureaucracy and an increased emphasis on making things happen’ (S‐Designated Nurse for Safeguarding) and ‘The pandemic acted as a catalyst to enhance partnership collaboration at both strategic and an operational level’ (S‐Safeguarding Partnership Business Manager). Adaptations to working practices, such as virtual meetings, greater information sharing and more frequent communication, resulted in more than half of respondents (56 per cent) agreeing or agreeing strongly that working relationships among the Safeguarding Partners and relevant agencies in their area improved. Although many respondents were neutral as to whether the quality of joint working between their own and other individual agencies had changed, they were more likely to report that joint working between their agency and another had improved than deteriorated, with respondents from all other agencies more likely to report improvements in strategic working with education than deterioration. In contrast, education respondents were more likely to report deterioration than improvement in joint working with all other agencies, both strategically and operationally. A few commentators warned, however, that increased communication does not necessarily equate with genuine collaboration: ‘there has [sic] been positives – more regular calls, and better attendance. but its [sic] been information sharing rather than how do we collaborate’ (S‐Safeguarding Partnership Business Manager). A survey respondent offered the following insight:

Whilst we have easier accessibility to partner agency professionals through virtual means actual partnership engagement with a focus on the needs of children has been impaired during the COVID period, professional/service need has in my view at times impaired a child centred approach to meeting the needs of families during these challenging times. (S‐Children's Social Care)

Interview participants described the wide variety and complexity of local joint agency arrangements, especially in the context of fragmented health services. Notwithstanding some concerns expressed as to the appropriateness, feasibility or successful achievement of tripartite responsibility, 70 per cent of survey respondents indicated that tripartite leadership and decision‐making had been successfully established or maintained during the pandemic. However, since successful tripartite leadership does not in itself guarantee improved joint working, we also asked whether the replacement of Local Safeguarding Children Boards with Safeguarding Partnerships was considered to have improved inter‐agency collaboration. While nearly half (49 per cent) of respondents expressed a neutral view, 39 per cent agreed or strongly agreed, with positive responses more common from police, children's social care and Safeguarding Partnership respondents. Data from the interviews and qualitative comments reveal some unease, including that the shift had resulted in ‘losing a great deal of the strength of the wider partnership’ (I‐Safeguarding Partnerships‐8) and that shrinking of the executive board could reduce operational impact. I‐Health‐15 observed: ‘you really need that operational input … there is now I would say, disconnect between that group of people [the executive], and the ones who work together to share operational information’. Exceptionally strong support was exhibited for greater involvement of relevant agencies in the work of Safeguarding Partnership subgroups: 98 per cent for education providers and CAMHS respondents, 96 per cent for health providers and 94 per cent for housing. A perhaps surprising level of support was also demonstrated for greater representation of these groups at Safeguarding Partnership executive boards (94 per cent for education providers, 88 per cent for health providers, 84 per cent for CAMHS and 73 per cent for housing).

In some instances of local changes, such as unilateral decisions by different acute trusts to withdraw midwifery home visits, more active collaboration between provider leads and Safeguarding Partnerships might have alleviated the impact on other agencies and on families. Survey respondents were strongly in favour (82 per cent) of the proposal that redeployment of safeguarding lead staff be agreed by Safeguarding Partnerships and largely in favour (72 per cent) of similar arrangements in relation to universal health staff. They also exhibited overwhelming support (91 per cent) for the proposal that future plans for redeployment of universal staff should be made in conjunction with safeguarding leadership within the relevant agency.

A wider issue in the context of the pandemic related to nationally or locally imposed decisions on which Safeguarding Partnerships were not consulted but which impacted critically on multiagency practice. Interviewees cited NHS England guidance as well as organisation‐level decisions on the redeployment of named and designated safeguarding professionals, health visitors and school nurses, and closure of children's centres by local authorities. Examples from survey respondents included the following:

Information sharing and partnership working at a local level has been improved by the response to the pandemic and in particular more flexible ways of working and technology. Challenges have mainly been created by decision[s] that are outside of local control particular[ly] in the health sector created by the tension caused by NHS national command and control culture. (S‐Children's Social Care)

The Local authority unilaterally paused clinical activity for Health visitors … [including] new birth visit for first time parents …A … baby died and investigation is ongoing. A number of babies also suffered Non accidental injury. (S‐Designated Nurse for Looked After Children)

## DISCUSSION

The innate reliance of safeguarding practice on multidisciplinary and inter‐agency work poses unique challenges in the arenas of leadership and organisational culture. Safeguarding is not always regarded as the core business of most of the organisations and agencies upon which children rely for their protection. The pandemic exacerbated an inherent tendency for safeguarding to be side‐lined as the focus of public policy and the delivery of health services shifted abruptly to prioritise containing the spread of the virus and treating its victims. A number of agencies, including children's social care, CAMHS and health visiting, were operating under significant pressure before the outbreak, which struck at a time when demographic changes had caused the focus of policy to be drawn towards adult social care.

Use of the six practice themes identified by the Panel is helpful in shedding light on the formidable barriers facing professionals, both in understanding the changing environments in which children were living and in identifying and assessing new or altered risks due to the pandemic. Direct engagement with children and families was dramatically reduced, while families' opportunities to evade professional scrutiny with impunity and for passive disengagement appear to have significantly increased. Well‐established pathways and fora for the sharing of information and collaborative relationships between professionals were disrupted by redeployment, self‐isolation and home‐working arrangements. Professional curiosity and professional challenge were diminished by high workloads, stress, exhaustion and burnout. We have yet to fully understand the price that will be paid in terms of safeguarding. Sadly, however, forthcoming local and national reviews appear likely to substantiate our findings. Changes in referrals to children's social care are difficult to interpret: reduced referral rates (DfE, 2021) are much more likely to have been the consequence of lost opportunities to identify children at risk than to reflect improvements in children's lives, and the reported increases in the complexity and severity of referrals may indicate multiplication of risk factors affecting children and/or delayed identification. Despite the many initiatives taken to tackle the impediments to effective safeguarding during the pandemic recounted above and evidence that referrals have increased in severity and complexity, fewer public law cases were received by CAFCASS between April 2020 and March 2021 compared with the preceding year (CAFCASS, 2021). This suggests that a surge in public law applications may be yet to come at the time of writing.

Nonetheless, the findings reported here illustrate a number of opportunities for enhanced multiagency working and inter‐agency collaboration. The crisis appears to have accelerated the embedding of new Safeguarding Partnership arrangements and strengthened the appetite for collaborative engagement at local level, a finding endorsed by Wood's report into the operation of Safeguarding Partnerships (Wood, 2021). Virtual communication offers promise for improvements in efficiency and engagement at multiagency fora. Heightened anxieties over rapidly changing home circumstances for children may have influenced the very high levels of support that the study elicited for enhancements in information sharing. Nonetheless, renewed consideration should be given to greater use of shared information databases. One possibility would be to extend the Child Protection – Information Sharing project (NHS Digital, 2021), which is currently limited to children on a child protection plan or looked after, and operates between health and social care only, although it was extended to school nurses and health visitors during the pandemic. Collaborative or shared risk assessments provide potentially fruitful areas for further development: the Panel's anticipated thematic review of risk assessment and decision‐making will provide timely insight.

Although the study was not able to investigate the operation of Safeguarding Partnerships in detail, it is clear that experiences during the pandemic have illuminated the importance of partnerships engaging relevant agencies fully in their work. In particular, it draws attention to the importance of engaging schools, as the sector with the most interaction with children and families, in Safeguarding Partnership arrangements. In this regard, the DfE's request that Safeguarding Partnerships review their engagement with schools is welcome. Another question which arises is how integrated care systems (recently introduced partnerships for the coordination of health and social care needs at local area level, including with the voluntary, community and social enterprise sector) will interact with Safeguarding Partnerships.

## CONCLUSION

Our study provides a unique perspective on the operation of the child protection system during the Covid‐19 pandemic through the insights of leaders from a range of professional disciplines and agencies. These have enabled us to shed light on some of the complexities of inter‐agency working in an unprecedented context and on the lessons for effective multiagency leadership which have emerged as Safeguarding Partnerships become more established.

Although the nature and consequences of each emergency will differ, the pandemic has revealed the critical importance of contingency planning within and across all sectors to determine how services should best be reorganised to ensure the emergency response does not inflict greater harm than good on some populations. At local area level, Safeguarding Partnerships are ideally positioned to lead both on drawing up plans to protect the vital mechanisms and pathways for identification of safeguarding concerns and coordination of child protection responses, and on liaising with all agencies to ensure that single‐agency decision‐making does not disrupt the complex web of collaboration that underpins safeguarding practice.

Two further areas of concern stand out as having been greatly exacerbated by the crisis. The first of these relates to the provision and accessibility of early help services, which elicited strong calls for greater investment by those taking part in the study and was identified as an area of concern by the Independent Review of Children's Social Care (2021). One of the study's most significant findings is the way in which relationships with families improved during the pandemic in response to actions which were seen as supportive rather than threatening. Investment in early help is imperative to shift practice from a punitive, crisis‐focused approach to the model of partnership with families envisaged by the Children Act 1989. The second and related issue concerns safeguarding staff capacity. The extraordinary dedication of safeguarding professionals is abundantly clear from our data, but so too is the impact of the pandemic on professional wellbeing. Skilled and supported staff are fundamental to effective safeguarding practice. As safeguarding professionals from all disciplines brace themselves for increased levels of need in the aftermath of the pandemic, reprioritisation of children's wellbeing in national and local policy and effective multiagency working will be ineffectual if it is not underpinned by attention to professional capacity and wellbeing across disciplines.

## CONFLICT OF INTEREST

No conflicts of interest arise.

## ETHICS STATEMENT

Ethical approval was granted by King's College London Research Ethics Committee.
